# Uncertainty about paternity: a study on deliberate ignorance

**DOI:** 10.3389/fpsyg.2024.1399995

**Published:** 2024-09-05

**Authors:** Gerd Gigerenzer, Rocio Garcia-Retamero

**Affiliations:** ^1^Max Planck Institute for Human Development, Berlin, Germany; ^2^Department of Experimental Psychology, University of Granada, Granada, Spain

**Keywords:** anticipated regret, deliberate ignorance, DNA paternity tests, Germany, insurance, paternity, risk aversion, Spain

## Abstract

Deliberate ignorance is the willful choice not to know the answer to a question of personal relevance. The question of whether a man is the biological father of his child is a sensitive issue in many cultures and can lead to litigation, divorce, and disinheritance. Thanks to DNA tests, men are easily able to resolve the uncertainty. Psychological theories that picture humans as *informavores* who are averse to ambiguity suggest men would do a DNA test, as does evolutionary theory, which considers investing in raising a rival’s offspring a mistake. We conducted two representative studies using computer-based face-to-face interviews in Germany (*n* = 969) and Spain (*n* = 1,002) to investigate whether men actually want to know and how women would react to this desire. As a base line, Germans (Spanish) estimated that 10% (20%) of fathers mistakenly believe that they are the biological father of their child. Nevertheless, in both countries, only 4% of fathers reported that they had performed a DNA paternity test, while 96% said they had not. In contrast, among men without children, 38% (33%) of Germans (Spanish) stated they would do a DNA test if they had children, mostly without telling their partners. Spanish women with children would more often disapprove of a paternity test or threaten their husbands with divorce (25%) than would German women (13%). We find that a simple test of risk aversion, measured also by the purchase of non-mandatory insurances, is correlated with not wanting to know.

## Introduction

In August Strindberg’s (2014) *The Father*, a cavalry captain learns that he is not the father of the daughter he adores. Without a biological link, he laments, paternal love is without foundation. He finds consolation in his childhood nursemaid and, his head nestled in her lap, speaks of the comfort of his “mother,” the role the nursemaid assumed for him. Strindberg’s play seizes on the conflicting forces of biological and social paternity.

The question of whether a man is the biological father of his child is a sensitive issue in many cultures and can lead to litigation, divorce, disinheritance, and disputes about child support (Anderson et al., 2007). Because of internal fertilization and live birth, a human female can be practically certain about her biological parenthood, whereas a male has to live with the uncertainty that someone else might be the biological father of his child. Until recently, men had to rely on uncertain cues such as physical resemblance or ABO blood tests that could exclude but not prove paternity. Modern DNA technology (“genetic fingerprinting”) can resolve this uncertainty with practical certainty. Paternity is typically concluded if the probability that two individuals are biologically parent and child is estimated at 99.99% or higher. The necessary material is easy to obtain (mouth swab, hair with roots, or used Kleenex), and the test is relatively cheap, approved by courts, and available for purchase on the internet. Now that paternal certainty is only “one click away,” do men want to find out?

In this article, we begin with theoretical perspectives that suggest different answers to the question. Then we report the first nation-wide representative studies in two large European countries, Germany and Spain, where we asked men with (without) children whether they had performed (would perform) a DNA paternity test, and women with (without) children about how they would react if their husband or partner asked for a DNA test. To see whether not wanting to know is associated with risk aversion, as it has been reported in previous studies on deliberate ignorance, we conducted a standard risk aversion test and also obtained data about real-life risk aversion as expressed by purchasing non-mandatory insurances.

## The case for wanting to know

Much of philosophy and psychology has assigned a positive value to the power of knowing, and sometimes deemed it a moral obligation. Aristotle began his *Metaphysics* (Aristoteles, 1953) with the dictum “All men by nature desire to know.” Locke (1690/1953) listed ignorance as the first cause of wrong judgment. Logical positivists such as Rudolf Carnap (1969) argued that valid information should not be left on the table, and Bayesian statisticians such as I. J. Good (1967) reasoned that one’s prior probabilities should be updated by new information. Similarly, modern psychological theories on information search assume that people want to know. Psychological theories generally picture humans as *informavores* (Miller, 1983) who are averse to ambiguity (Hogarth, 1987) and in need of closure (Kruglanski and Webster, 1996). Likewise, most theories in neo-classical economics assume that rational choice requires all relevant information to be known, and if not, actively searched for, until the costs of search exceed its expected benefits (Rizzo and Whitman, 2020). The desire for information appears to be the natural condition of humankind, whereas not wanting to know seems irrational and has often been linked to self-deception and shirking responsibility, as when women refuse to participate in breast cancer screening and people at risk for HIV do not pick up their test results (Thornton, 2008; Hertwig and Engel, 2020).

Given that the ability to invest in children is a limited resource, evolutionary theories focusing on inclusive fitness arrive at a similar conclusion. Altruistic behavior such as parental investment is assumed to be proportional to the genetic relatedness between donor and recipient (Hamilton, 1964; Alexander, 1974; Trivers, 1974; Anderson, 2006). These various *parental investment theories* predict that men’s investment in children is a function of their confidence in paternity. In the words of Daly and Wilson (2006), “From the gene’s eye view, laboring to raise a rival’s offspring is a disastrous mistake” (p. 195), and “we might therefore expect men to be sensitive to available information about paternity” (p. 196).

In this view, a man can make two kinds of error: invest in a rival’s offspring because he mistakenly believes himself to be the biological father or invest in his own child insufficiently because he mistakenly suspects that he is not the biological father. In terms of signal detection theory, the first error is a false positive, the second a miss. Today, a DNA paternity test can reduce both errors to practically zero. Thus, various philosophical, psychological, and biological theories converge to the conclusion that it is rational for men to do a DNA test in order to eliminate paternal uncertainty.

## The case for not wanting to know

Research on *deliberate ignorance* has documented cases where the expected desire for information does not hold and a substantial proportion of people willfully remain uninformed. For instance, after East Germany’s Stasi records were opened in 1991, many citizens declined the opportunity to read their personal files. In their seminal analysis, Hertwig and Ellerbrock (2022) estimated that although about 40% of adult citizens believed that a Stasi file on them existed, more than half of these did not access it. Interviews uncovered a variety of reasons for this choice, including the anticipation of negative emotions and personal conflict if personal files were to reveal colleagues, friends, or family members who had spied on them (Hertwig and Ellerbrock, 2022).

The concept of deliberate ignorance refers to the willful decision not to know, as opposed to the inability to access information or mere disinterest. Deliberate ignorance requires two conditions (Gigerenzer and Garcia-Retamero, 2017, p. 180):

1. Choice of ignorance even when information is free or search costs are negligible.
2. Choice of ignorance notwithstanding personal interest.

Thus, deliberate ignorance is neither a result of another party withholding information nor the result of indifference or forgetting. Nor does it resemble a search for confirmatory information, as studied in the selective exposure literature (see Sweeny et al., 2010). The study of deliberate ignorance is also to be distinguished from the study of agnotology (Proctor and Schiebinger, 2008) and the sociology of ignorance (McGoey, 2014), which investigate the systematic production of ignorance by obscuring knowledge or disseminating fake news, as in generating and supporting public ignorance about global climate change.

Four key motives for deliberate ignorance have been identified (Hertwig and Engel, 2020; Gigerenzer and Garcia-Retamero, 2017). Three of these do not apply to the present study: achieving fairness and impartiality (as embodied by blindfolded Lady Justice), gaining strategic advantage (as in bankers’ willful blindness to risks that led to the financial crisis of 2008; see Admati and Hellwig, 2013), and suspense and surprise (e.g., 40% of Germans do not want to know the sex of their child before birth, and instead wish to maintain the suspense and surprise; Gigerenzer and Garcia-Retamero, 2017).

The fourth motive is relevant for the present study: to avoid potentially bad news and subsequently regret having to live with it, particularly in situations that one cannot change. For instance, when agreeing to have his genome sequenced, James Watson, the co-discoverer of DNA, requested that information about his ApoE4 genotype, which indicates risk of Alzheimer’s disease, be deleted from his published genome and not revealed to himself (Wheeler et al., 2008). Watson had perhaps concluded that because the disease is incurable, the anticipated regret of living with bad news would be larger than the meager benefits of knowing (Hertwig and Engel, 2020). The decision of many citizens not to read their personal Stasi records is another case in point. This motive is known as *anticipatory regret*.

*Regret* is a negative emotion that people may experience *after* choosing option A (e.g., not buying fire insurance) and later learning that option B (buying insurance) would have resulted in a more favorable outcome. *Anticipated regret* is an emotion that occurs *before* the choice is made (Luce and Raiffa, 1957). Anticipating possible regret may itself influence the choice. One imagines what would happen if an outcome were known and then decides not to know.

For the present topic, men might prefer deliberate ignorance because they anticipate regret about having performed a DNA test. If the test shows non-paternity, they might regret facing this new situation, in particular, their relation with spouse and child; if the test confirms paternity, they might regret having done the test and offended their partner by mistrusting her. Thus, to the degree that men have anticipatory regret, they should prefer deliberate ignorance about paternity.

The regret theory of deliberate ignorance (Gigerenzer and Garcia-Retamero, 2017) is based on Luce and Raiffa’s (1957) classical regret theory and makes several general predictions, which are formally derived in Gigerenzer and Garcia-Retamero (2017). The first is that anticipatory regret increases the nearer the event is, that is, the nearer regret can occur.

### Are men with or without children more likely to consider a paternity test?

According to parental investment theories, men with children should be most interested in doing a paternity test, while theories that picture humans in general as informavores do not make a specific prediction, so men without children might be equally interested in doing a test if they were a parent. In contrast, the regret theory of deliberate ignorance specifically predicts that the closer in time to the critical event that could generate regret, the higher the anticipated regret and the lower the number of individuals who want to know (Gigerenzer and Garcia-Retamero, 2017). For instance, the older people are, the less likely they want to know when they and their partner will die (Gigerenzer and Garcia-Retamero, 2017). This dependence of the rate of deliberate ignorance in a population on the time to the possible regret is the *time-to-event hypothesis.* In the case of paternity, it leads to this prediction:

Prediction 1: *Men without children are more likely to say that they would want to know, whereas those who have children are less inclined to actually find out.*

The rationale is that men without children can less likely imagine the anticipated regret of knowing than can men with children.

## Risk aversion

The regret theory of deliberate ignorance is a direct extension of Luce and Raiffa’s (1957) regret theory, which was formulated for risky choices. It facilitates deducing predictions about the relation between risk aversion and deliberative ignorance (Gigerenzer and Garcia-Retamero, 2017). Here we apply this theory for the first time to uncertainty about paternity.

### Risk aversion test

People are said to be risk averse for gains if they choose a certain gain *v* = $X over a gamble with a higher expected gain. To measure risk aversion, we used a standard paradigm, where participants can choose between a sure gain and a gamble. The rationale for Prediction 2 lies in the asymmetry of the possibility of the experience of regret in the standard risk aversion paradigm. If the risky gamble is chosen, it is played out and regret can occur if the result is less than the certain gain. If the certain gain is chosen, the risky gamble is not played out, meaning that it is not possible to know whether choosing the risky option would have led to a better or worse outcome. Regret is possible only when people choose the risky option and the result is unfavorable. By selecting the certain gain, an individual can thus avoid regret.

In other words, the same motivation—avoiding anticipatory regret—underlies both risk aversion and deliberate ignorance. Hence, we predict that if deliberate ignorance is due to regret avoidance, it should be more frequent among men who are risk averse.

Prediction 2: *Men who are risk averse for gains are more likely to exhibit deliberate ignorance.*

Consider now losses. People are said to be risk averse for losses if they choose a certain loss *v* = $X over a gamble with a smaller expected loss. As explained above, regret is only possible if a person chooses the risky option. Thus, by choosing the certain loss, one can avoid the possibility of regret.

*Prediction* 3*: Men who are risk averse for losses are more likely to exhibit deliberate ignorance.*

Note that Predictions 2 and 3 assume that risk aversion applies to both gains and losses, unlike the hypothesis that people are risk averse for gains and risk seeking for losses (Kahneman and Tversky, 1979).

### Purchasing non-mandatory insurance

Buying non-mandatory insurance such as life and property insurance is equivalent to choosing a sure loss *v* = $X (the insurance premium) over a probable loss with a lower expected loss. Thus, buying non-mandatory insurance is equivalent to risk aversion for losses, which leads to the following prediction:

Prediction 4: *Men who buy non-mandatory insurance are more likely to exhibit deliberate ignorance.*

Note that the predictions state correlations between deliberate ignorance and measures of risk aversion, including purchasing non-mandatory insurance, not causations. We also do not postulate that the two feelings—anticipatory regret in the case of paternity and risk aversion in the case of gambles and insurance—are of the same subjective quality or currency, but only that they are correlated. To the best of our knowledge, Predictions 1 to 4 are new and have never been tested in the context of paternity uncertainty. Confirming them would provide support for the regret theory of deliberate ignorance. Moreover, it would demonstrate that the classical measure of risk aversion is a valid diagnostic test for men’s attitudes toward wanting to know about paternity.

## Women’s willingness to agree

Women’s reaction to their partners’ request for a paternity test likely depends on the cultural context. One might thus expect differences between the two countries, but it was not clear to us in which direction. Risk aversion, in contrast, allows for a prediction. If the classical risk test has diagnostic power, risk-averse women should more likely agree to their partners’ request for a paternity test than risk-seeking women. For instance, women with small children may be financially dependent on their partners and might anticipate that openly disagreeing with the request would only heighten their partner’s suspicion and endanger emotional and financial support. In this way, they might anticipate regret for having disagreed openly.

We measured risk aversion for women in the same two ways as for men, by a classical risk aversion test and by the possession of non-mandatory insurance.

## Method

### Population and sample

We hired the international survey company GfK Group, based in Nuremberg, Germany, with an office in Valencia, Spain. GfK obtained nationwide quota samples of 1,016 adults in Germany and 1,002 adults in Spain. The samples were representative of the population in each country in terms of four variables: age, gender, region, and size of settlement. In the German sample, 47 participants did not complete the questions, which reduced the sample size to 969. Table 1 shows the characteristics of the two samples. The paternity study was part of a larger survey on deliberate ignorance (Gigerenzer and Garcia-Retamero, 2017). We report 95% confidence intervals (CIs) for sample statistics. When 95% CIs are used, our sample size of approximately 1,000 participants per country provides a power of 0.99 to detect a small effect size (corresponding to Cohen’s *h* = 0.2) and a power of over 0.995 to detect a medium effect size (corresponding to Cohen’s *h* = 0.5; Cohen, 1988). The ethics committee of the Max Planck Institute for Human Development approved the methodology.

**Table 1:** The German and the Spanish sample by gender, age, religious practice, education, marital status, risk aversion, and non-mandatory insurances bought.

	Germany		Spain	
	*n*	%	*n*	%
Total	969	100.0	1,002	100.0
Gender				
Male	471	48.6	491	49.0
Female	498	51.4	511	51.0
Age				
18–35	306	31.6	322	32.1
36–50	294	30.3	304	30.4
51+	369	38.1	376	37.6
Religious services per month				
0 times	683	70.5	699	69.8
1–2 times	187	19.3	175	17.5
3+ times	99	10.2	127	12.7
Education				
1	45	4.7	53	5.3
2	374	39.3	139	14.0
3	317	33.3	328	32.9
4	129	13.6	321	32.2
5	87	9.1	155	15.6
Marital status				
Married	391	40.4	409	40.8
Not married	578	59.6	593	59.2
Risk aversion				
1	216	31.8	271	35.9
2	283	41.6	132	17.5
3	154	22.6	287	38.0
4	27	4.0	65	8.6
Insurance				
Life	575	59.3	423	42.2
Household	757	78.1	712	71.1
Personal	751	77.5	227	22.7
Legal	440	45.4	51	5.1

Education: 1 = primary/lower secondary school without vocational training; 2 = primary/lower secondary school with vocational training; 3 = further education without secondary school leaving qualification (US: high school diploma); 4 = secondary school leaving qualification; 5 = university. Percentages do not add up to 100% because 18 Germans and 6 Spaniards were still in school. Risk aversion: 1 = risk averse for gains and risk seeking for losses; 2 = risk averse for gains and losses; 3 = risk seeking for gains and losses; 4 = risk seeking for gains and risk averse for losses (numbers do not add up to total sample size because risk neutrals are not included). Insurance: Life = life insurance; Household = household insurance (“Hausratsversicherung”); Personal = personal liability insurance (“Privathaftpflicht”); Legal = legal expenses insurance.

### Procedure

To ensure the quality of data for this sensitive topic, we invested in computer-based face-to-face interviews and risk aversion tests rather than a less expensive telephone or internet survey. After a first telephone contact was established, all participants were interviewed individually in their homes. Participants could enter their responses directly into the computer. To begin with, they were asked to estimate the frequency of non-paternity in their countries. Males were asked whether they had performed DNA testing or, for those who did not have children, whether they intended to perform DNA testing when they had children. Females were asked about their reactions to their partner’s wanting to know.

All participants took two tests of risk aversion, one for gains and one for losses.

*Risk aversion for gains*:

You won a contest and have to choose between two alternatives: a lottery and a sure gain. The lottery has 10 items, five of which win 100 euros, the others nothing. Would you prefer the sure gain to the lottery?

Win 20 euros for sure instead of the lottery. yes/no

Win 30 euros for sure instead of the lottery. yes/no

Win 40 euros for sure instead of the lottery. yes/no

Win 50 euros for sure instead of the lottery. yes/no

Win 60 euros for sure instead of the lottery. yes/no

Win 70 euros for sure instead of the lottery. yes/no

The interviewer presented the options, in the order shown above, successively to the participant on a computer screen until the participant answered “yes.” If a person preferred a sure gain to a lottery with a higher expected value, they were classified as risk averse for gains. An example would be a participant who preferred a sure win of 40 euros to a lottery whose expected value is 50 euros. If a person preferred a lottery to a sure gain despite the lottery having a smaller expected gain, they were classified as risk seeking for gains. An example would be a participant who preferred the lottery to a sure win of 60 euros.

*Risk aversion for losses*:

You lost a contest and have to choose between two alternatives: a lottery and a sure loss. The lottery has 10 items, for five of which you have to pay 100 euros, for the others nothing. Would you prefer the sure loss to the lottery?

Pay 70 euros for sure instead of the lottery. yes/no

Pay 60 euros for sure instead of the lottery. yes/no

Pay 50 euros for sure instead of the lottery. yes/no

Pay 40 euros for sure instead of the lottery. yes/no

Pay 30 euros for sure instead of the lottery. yes/no

Pay 20 euros for sure instead of the lottery. yes/no

A person is said to be risk averse for losses if preferring a sure loss to a lottery with a smaller expected loss, and risk seeking for losses if preferring a lottery to a sure loss when the lottery has a higher expected loss.

## Results

### Perceived prevalence of non-paternity

We obtained base rate estimates from both the German and the Spanish sample regarding their perceived rate of non-paternity:

What is your estimate of how many fathers in Germany [Spain] mistakenly believe that they are the biological father of their child?

_____ out of every 1,000.

Among Germans, the average estimate was 96 in 1,000; among Spaniards, it was twice as high, 199 in 1,000. Thus, the general public in both countries appears to understand the potential magnitude of this eventuality. The average estimates of 10 and 20% are at the high end of scientific estimates reported in the literature, with the caveat that objective figures are hard to obtain (see Discussion Section).

### Do fathers want to know?

Next, we asked men with children:

A DNA test can determine paternity with high certainty. All one needs is a hair from the child’s head. Have you ordered a test of one or more of your children to be sure that you are the biological father?

Only 4% (4%) of the men with one child in Germany (Spain) said that they had done a paternity test. Among men with several children, the proportion was similar, 4% (5%). In contrast, 96% of fathers reported not having done a DNA test.

We found that those who said they had performed or would perform a DNA test had higher estimates of non-paternity. Among Germans, the average estimate in this group was 106 in 1,000 (SEM = 5.3) compared with 91 in 1,000 (SEM = 5.3) among those who would not perform or had not performed the test. The same pattern occurred among Spaniards, with estimates of 224 in 1,000 (SEM = 10.1) and 179 in 1,000 (SEM = 8.9), respectively. This association could indicate that beliefs about the frequency of non-paternity influence the decision about conducting a paternity test, but it could also mean that the decision influences the estimates.

In sum, 96% of men in both countries with children reported that they had not performed a DNA paternity test to determine whether or not they were the biological father of their children.

### Men with and without children

According to Prediction 1, men without children are more likely to say that they would want to know, whereas those who have children are less inclined to actually find out. To test this, we asked men without children the same question as for men with children, except that it was phrased “Assume you are married and have a 3-year-old child. A DNA test can determine paternity with high certainty. All one needs is a hair from the child’s head. Would you order…”

**Figure 1:**
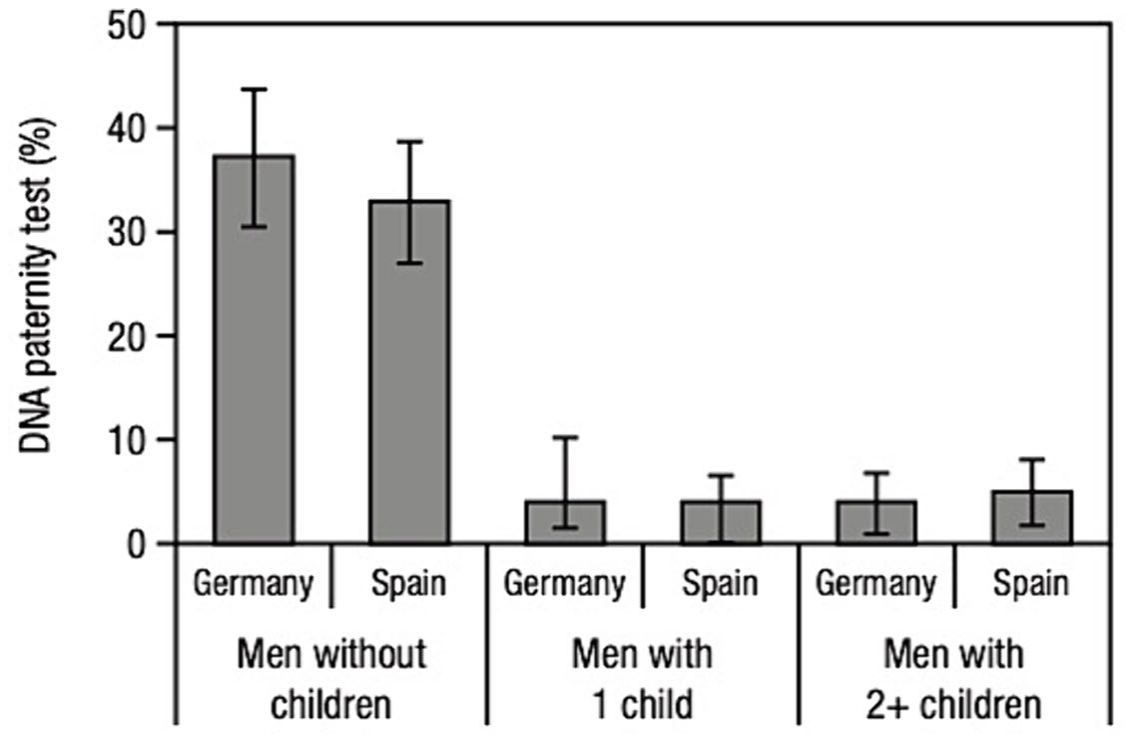
Time-to-event hypothesis. The reported intention to perform a DNA paternity test is relatively high among men without children in both Germany and Spain, while fathers’ reported frequency of actual paternity tests is comparably low. The bars show the 95% confidence intervals for the point estimates. Data from national representative quota samples of German (*n* = 439) and Spanish (*n* = 491) men.

Thirty-eight percent of German men and 33% of Spaniards without children answered “yes” (Figure 1, left two panels). This substantial difference to the average 4% of fathers who reported that they had actually done a DNA test (Figure 1, right four panels) is consistent with the time-to-event hypothesis (Prediction 1), but not with the hypothesis of an increasing desire to know. We checked whether this effect could be due to the age difference between those with and without children, with the former being on average older. But, consistent with the hypothesis, a logistic regression analysis using all variables in Table 1 showed that age was not a valid predictor, whereas marital status and having children were.

With respect to honesty, the majority of men without children who said that they would conduct a DNA test indicated that they would not tell their wives. Among Germans, the 38% figure splits into 23% who would not inform their wives and 15% who would; among Spaniards, the 33% figure splits into 21 and 12%, respectively. Note that “secret” paternity testing without both parents’ full consent is illegal in Germany under the Gene Diagnostics Act of 2009. The current Spanish law also requires consent, although it does not specify what happens if the mother does not consent (Barrot et al., 2014).

### Risk aversion

Previous research reported that deliberate ignorance is more frequent among people who are risk averse than among those who are risk seeking. This phenomenon was observed in contexts other than paternity, both for negative events such as wanting to know the time one will die and for positive events such as wanting to know what presents one will get for Christmas (Gigerenzer and Garcia-Retamero, 2017). Does a similar association hold for paternity as well?

Table 2 reports the results aggregated across the two countries and across men with and without children because these were consistent. Among men who were risk averse for gains, 85.3% (422 of 495) stated they did not want to know, compared with 76.6% among those classified as risk seeking, resulting in a difference of 8.7 percentage points (95% CI = 2.9–14.4). This result is consistent with Prediction 2. Among men who were risk averse for losses, the difference is also consistent with Prediction 3, but smaller in size, with a difference of 2.7 percentage points and the confidence interval including zero. Across both risk attitudes, gains and losses, deliberate ignorance is 5.3 percentage points higher among risk-averse men (95% CI = 1.5–9.0). Thus, overall, risk aversion among men is associated with not wanting to know about paternity.

**Table 2:** Risk aversion and purchase of non-mandatory insurance are diagnostic for men’s choice of deliberate ignorance about biological paternity.

Proportion of men (*n/N*) who do not want to know			
Risk attitude	Risk averse	Risk seeking	Difference (risk averse – risk seeking) in percentage points [95% CI]
Gains	85.3% (422/495)	76.6% (229/299)	8.7 [2.94 to 14.39]
Losses	83.6% (225/269)	81.0% (439/542)	2.6 [−2.87 to 8.16]
Total (gains + losses)	84.7% (647/764)	79.4% (668/841)	5.3 [1.52 to 9.00]
Purchase of non-mandatory insurance	Insurance	No insurance	Difference (Yes – No) in percentage points [95% CI]
Life insurance	85.2% (403/473)	78.1% (382/489)	7.1 [2.22 to 11.95]
Property insurance	85.5% (572/669)	72.7% (213/293)	12.8 [7.05 to 18.56]
Personal insurance	86.4% (412/477)	76.9% (373/485)	9.5 [4.61 to 14.32]
Legal insurance	90.9% (219/241)	78.5% (566/721)	12.4 [7.66 to 17.08]
Total (all insurances)	86.3% (1,606/1,860)	77.2% (1,534/1,988)	9.2 [6.76 to 11.60]

Men with and without children are included. *n* = number of men in the subgroup who did not want to know, *N* = total number of men in subgroup. For instance, among the 495 men who were risk averse for gains, 422 did not want to know (85.3%), while among the 299 men who were risk seeking for gains, only 229 (76.6%) did not want to know. Men who were neither risk averse nor risk seeking but risk neutral are not included.

### Purchase of non-mandatory insurance

We asked participants whether they had bought life, property, personal, and legal insurances (the four most frequent non-mandatory insurances in Germany and Spain). According to Prediction 4, men who buy these insurances are more likely to exhibit deliberate ignorance. This prediction is correct for each of the four insurances (Table 2). For instance, 85.2% of men who had bought life insurance said they did not want to know whether they are the biological father of their child, compared with 78.1% of men who had not purchased life insurance, resulting in a difference of 7.1 percentage points (95% CI = 2.2–11.9). Across all four insurances, the percentage who reported not wanting to know was 9.2 percentage points higher among men who had purchased insurance (95% CI = 6.8–11.6).

In sum, the tests of Predictions 1 to 4 were consistent with the regret theory of deliberate ignorance. That is, risk aversion, as measured by a simple risk test or by the possession of non-mandatory insurances, can serve as a diagnostic test of men’s willingness not to know about paternity.

### Women’ s reaction to husband’s request for a paternity test

How would women react if their husband or partner wanted to find out whether he is the biological father? We asked the female participants:

Assume you are married and have a 3-year-old child. A DNA test can determine paternity with high certainty. All one needs is a hair from the child’s head. Your husband wants to conduct a test to be sure that he is the biological father of the child. How would you react?

1 *I would agree because I have nothing to hide.*

German women without children: 50%; with children: 57%.

Spanish women without children: 35%; with children: 45%.

2 *I would agree but I would be offended.*

German women without children: 33%; with children: 30%.

Spanish women without children: 33%; with children: 30%.

3 *I would not agree.*

German women without children: 8%; with children: 9%.

Spanish women without children: 19%; with children: 16%.

4 *I would threaten with divorce or separation.*

German women without children: 8%; with children: 4%.

Spanish women without children: 13%; with children: 9%.

The responses reveal two major results. The first is a pattern similar to the time-to-event hypothesis (Figure 1). Women without children, in comparison to women with children, less often said they would agree and more often said they would threaten with divorce or separation. We checked whether this effect could be due to the age difference between those with and without children. As in the case of the men, a regression analysis using all variables in Table 1 showed that age was not a valid predictor but marital status and having children were. Thus, among couples with children, more women said they would tolerate men’s wish to be certain about paternity, while few men actually have this wish.

The second result is a cultural difference between German and Spanish women. About twice as many Spanish women with children would not agree to a paternity test or would threaten their husbands with divorce or separation (25%) than German women with children (13%). Correspondingly, more German than Spanish women said they would agree to testing because they had nothing to hide (a difference of 15 percentage points for women without children, and 12 percentage points for those with children). A regression analysis showed that culture remained a valid predictor when controlled for the other variables in Table 1.

Thus, Spanish women were less likely to accept paternity tests than their German counterparts, which may have to do with traditional values of honor and marital integrity, or also reflect more recent developments such as that Spain has surpassed Germany in the number of women in full-time employment, leadership positions, and active military service.

### Risk aversion and insurance

We compared women who would agree because they had nothing to hide (response alternative 1) with those who would be offended, not agree, or threaten with divorce or separation (other response alternatives), for short, “agree” versus “offended.” Women who were risk averse for gains were more likely to agree to a paternity DNA test than those who were risk seeking (Table 3). The difference was 12.1 percentage points (95% CI = 5.1–19.2). A similar difference replicates for women who were risk averse for losses. Across both gains and losses, the willingness to accept the husband’s request for a DNA test was 10 percentage points higher among risk-averse women (95% CI = 5.3–14.7).

**Table 3:** Risk aversion and purchase of non-mandatory insurance are diagnostic for women’s willingness to consent to a DNA paternity test.

Proportion of women (*n/N*) who would agree with a paternity test			
Risk attitude	Risk averse	Risk seeking	Difference (risk averse – risk seeking) in percentage points [95% CI]
Gains	52.3% (301/575)	40.2% (111/276)	12.1 [5.05 to 19.21]
Losses	55.7% (156/280)	45.0 (260/578)	10.7 [3.64 to 17.82]
Total	53.4% (457/855)	43.4% (371/854)	10.0 [5.29 to 14.72]
Purchase of non-mandatory insurance	Insurance	No insurance	Difference (Yes – No) in percentage points [95% CI]
Life insurance	49.1% (258/525)	46.5% (225/484)	2.7 [−3.50 to 8.78]
Property insurance	49.4% (395/800)	42.1% (88/209)	7.3 [−0.34 to 14.63]
Personal insurance	53.3% (267/501)	42.5% (216/508)	10.8 [4.61 to 16.82]
Legal insurance	53.2% (133/250)	46.1% (350/759)	7.1 [−0.05 to 14.12]
Total	50.7% (1,053/2,076)	44.8% (879/1,960)	5.9 [2.80 to 8.95]

*n* = number of women in subgroup who would agree to DNA testing, *N* = total number of women in subgroup. For instance, among the 575 women who were risk averse for gains, 301 would agree to a DNA test without being offended (52.3%), while among the 276 women who were risk seeking for gains, only 111 (40.2%) would do so. Percentages are rounded.

Buying non-mandatory insurance was also associated with women’s willingness to accept a paternity test, but the absolute effect size was smaller (Table 3). Across all four insurances, the willingness to accept the husband’s request for a paternity test was 5.9 percentage points higher among insured women (95% CI = 2.8–8.9).

In sum, risk aversion, as measured by a simple test or by the possession of non-mandatory insurance, is diagnostic of women’s willingness to agree. Women who said they would be offended, not consent to testing, or threaten with divorce were more likely risk seeking and had not purchased non-mandatory insurances. The effect sizes are quite substantial, up to 12 percentage points, and similar for men and women.

It is surprising that a simple test of risk aversion can capture so well the attitudes of both men and women toward paternity testing.

## Discussion

The present study addressed the phenomenon of deliberate ignorance—the decision not to know particular information of personal relevance despite low search costs. Contrary to cognitive theories that picture humans as informavores, 96% of fathers in Germany and Spain reported that they had not performed a DNA test, and thus did not want to know. This finding clashes with expectations from a spectrum of theories, from philosophy to evolutionary biology, that emphasize the value of knowledge and the dangers of not knowing. Why would so many men not want to know? We suggested anticipatory regret as one of the motivations, derived four predictions from regret theory (Luce and Raiffa, 1957; Gigerenzer and Garcia-Retamero, 2017), and found support for these. Deliberate ignorance is higher (1) for men with children than for men without children (time-to-event hypothesis), (2) for men who are risk averse for gains, (3) for men who are risk averse for losses, and (4) for men who buy non-mandatory insurances. The results indicate that risk aversion is diagnostic for deliberate ignorance regarding paternity.

We showed that risk aversion is also diagnostic for women’s willingness to agree to a DNA paternity test. Women who are risk seeking would more likely not consent and would threaten with divorce or separation. This consistent finding supports the interpretation that women, like men, try to avoid situations for which they anticipate regret.

### Alternative explanations

Surveys, even with nationally representative quota samples, cannot provide a unique answer to what motivates deliberate ignorance. But we can use the evidence to exclude some alternative explanations. The first is that men might believe that non-paternity is so rare that it is not worth the effort of conducting a DNA test. We can exclude this explanation on the basis of participants’ estimates that 10% (Germans) or 20% (Spaniards) of fathers mistakenly believe that they are the biological father of their children. In the literature, the frequency of actual non-paternity has also sometimes been estimated between 10 and 20% (e.g., Baker and Bellis, 1995; Gaulin et al., 1997; Alfred, 2002), but these figures appear to be inflated as estimates for the general population because of selection biases, such as mistrustful fathers visiting paternity-testing laboratories. A meta-analysis with more than 24,000 subjects from mostly Caucasian populations estimated a rate of 2–3% (Voracek et al., 2008). A study in Germany estimated the non-paternity rate as around 1% (Wolf et al., 2012). Whatever the true rates are, the participants in our study estimated the frequency of non-paternity at the high end. Thus, the explanation that men consider non-paternity a negligible phenomenon has little support.

A second explanation is that men might not believe that DNA testing is reliable. There might be too many misses and false alarms. To determine whether our participants were aware of the high accuracy of DNA profiling, we asked whether the result of a DNA test is “absolutely certain.” Seventy-eight percent of the participants in Germany and 89% in Spain thought so. This result is consistent with an earlier representative survey, where 78% of Germans also thought that the result of DNA test is absolutely certain, compared with 63% for fingerprints and HIV tests (Gigerenzer et al., 2007). The fact that the large majority believed that DNA tests are absolutely certain makes lack of trust in the reliability of the test an unlikely explanation for why so many men did not use the test.

Finally, we checked whether religion or education could explain men’s wanting to know and women’s willingness to accept. Religious belief was measured by the number of attendances of religious services per month (Table 1). A regression analysis using all variables in Table 1 showed that neither religion nor education was associated with whether men wanted to know, when controlled for other factors. The same result was obtained for women. That education made no difference may come as a surprise, yet it is consistent with studies of deliberate ignorance in other contexts (Gigerenzer and Garcia-Retamero, 2017).

The four hypotheses we tested were derived from the assumption that anticipatory regret is a key factor for deliberate ignorance. That is, a man imagines that after seeing the test result, he might wish he had not done the test. Anticipatory regret increases the closer one is to the point at which regret can occur. Regret can be avoided by being risk averse, as measured by the classic test for risk aversion, both for gains and for losses. Similarly, purchasing non-mandatory insurance is motivated by anticipatory regret. All of these factors proved to be diagnostic, suggesting that a key motivation for deliberate ignorance is anticipated regret, consistent with previous results in other domains (Gigerenzer and Garcia-Retamero, 2017).

### Strengths and limits

A unique strength of this study is that we obtained two representative quota samples from two countries and conducted the computer-assisted interviews in person by visiting the participants’ homes. The downside was the substantially higher cost of this survey method compared with telephone or internet surveys. We decided upon this more expensive and labor-extensive procedure to secure the quality of the data, given the sensitivity of the topic.

One limit of the present study is that it relies on reported behavior rather than on measurements of actual behavior. We sought to reduce potential reporting bias by using computer-based face-to-face interviews with guaranteed anonymity. Nevertheless, the true number of paternity tests could be larger than the self-reported cases if some men did not admit to testing. To check this possibility, we obtained estimates of the actual sales of DNA paternity tests, which are difficult to verify given the multitude of companies that sell them. The best estimates for Germany seem to be in the order of 30,000 tests per year (Hipp, 2007). In relation to the approximately 680,000 newborns per year, this amounts to 4–5% of children being tested, which is consistent with the self-reports. Another limit is that we do not explicitly deal with how a man’s decision depends on his trust in his wife and how other members of the family would be impacted by a positive DNA test. However, one can consider the negative impact on the family, especially on the child, as part of the anticipated regret. A final limitation is that these two representative studies allow generalization to the population of Germany and Spain, but not necessarily to different cultures.

### Can deliberate ignorance be rational?

For those who believe that more information is always better, the majority of the men in both countries decide irrationally. As mentioned at the beginning of this article, philosophers such as Rudolf Carnap and Bayesian statisticians such as I. J. Good have proposed principles of rationality that imply one should not leave information on the table if it costs little or nothing. Anticipatory regret, in contrast, provides a reasonable explanation of this seemingly irrational behavior. Many do not want to know information that could become a disturbing problem. In the case of paternity, men’s decision not to know provides protection for the wellbeing of the children and the family, preferring trust to the objective potential of technology.

According to Greek mythology, Cassandra, the daughter of the king of Troy, was cursed by Apollo to foresee the future. Cassandra foresaw the death of her father, the hour of her own death, and the name of her murderer. If she had had the choice to stay deliberately ignorant, she would have been spared a life of incessant pain and suffering. Those of us who have that option can decide not to know. The logic of deliberate ignorance is to avoid the regret of knowing the worst possible outcome and to instead learn to live with uncertainty.

## Data availability statement

The raw data supporting the conclusions of this article will be made available by the authors, without undue reservation.

## Ethics statement

The studies involving humans were approved by the Ethics Committee Max Planck Institute for Human Development. The studies were conducted in accordance with the local legislation and institutional requirements. The participants provided their written informed consent to participate in this study.

## Author contributions

GG: Validation, Supervision, Project administration, Investigation, Formal analysis, Conceptualization, Writing – review & editing, Writing – original draft. RG-R: Writing – review & editing, Writing – original draft, Software, Methodology, Data curation.
